# Mitochondrial genome-wide association study of migraine – the HUNT Study

**DOI:** 10.1177/0333102420906835

**Published:** 2020-02-14

**Authors:** Sigrid Børte, John-Anker Zwart, Anne Heidi Skogholt, Maiken Elvestad Gabrielsen, Laurent F Thomas, Lars G Fritsche, Ida Surakka, Jonas B Nielsen, Wei Zhou, Brooke N Wolford, Magnus D Vigeland, Knut Hagen, Espen Saxhaug Kristoffersen, Dale R Nyholt, Daniel I Chasman, Ben M Brumpton, Cristen J Willer, Bendik S Winsvold

**Affiliations:** 1 Research and Communication Unit for Musculoskeletal Health, Division of Clinical Neuroscience, Oslo University Hospital, Ullevaal, Oslo, Norway; 2 Institute of Clinical Medicine, Faculty of Medicine, University of Oslo, Oslo, Norway; 3 K. G. Jebsen Center for Genetic Epidemiology, Department of Public Health and Nursing, Faculty of Medicine and Health Sciences, Norwegian University of Science and Technology, Trondheim, Norway; 4 Department of Neurology, Oslo University Hospital, Oslo, Norway; 5 Department of Clinical and Molecular Medicine, Norwegian University of Science and Technology, Trondheim, Norway; 6 HUNT Research Centre, Department of Public Health and General Practice, Norwegian University of Science and Technology, Levanger, Norway; 7 Department of Biostatistics, University of Michigan School of Public Health, Ann Arbor, MI, USA; 8 Department of Internal Medicine, Division of Cardiology, University of Michigan Medical School, Ann Arbor, MI, USA; 9 Department of Computational Medicine and Bioinformatics, University of Michigan, Ann Arbor, MI, USA; 10 Department of Medical Genetics, Oslo University Hospital, Oslo, Norway; 11 Department of Neuromedicine and Movement Science, Faculty of Medicine, Norwegian University of Science and Technology, Trondheim, Norway; 12 Norwegian Advisory Unit on Headache, Department of Neurology and Clinical Neurophysiology, St. Olavs University Hospital, Trondheim, Norway; 13 Department of Neurology, Akershus University Hospital, Lorenskog, Norway; 14 Department of General Practice, Institute of Health and Society, University of Oslo, Oslo, Norway; 15 School of Biomedical Sciences, Faculty of Health, Institute of Health and Biomedical Innovation, Queensland University of Technology, Brisbane, QLD, Australia; 16 Division of Preventive Medicine, Brigham and Women’s Hospital, Boston, MA, USA; 17 Harvard Medical School, Boston, MA, USA; 18 Department of Human Genetics, University of Michigan Medical School, Ann Arbor, MI, USA

**Keywords:** Migraine, mitochondria, HUNT

## Abstract

**Background:**

Variation in mitochondrial DNA (mtDNA) has been indicated in migraine pathogenesis, but genetic studies to date have focused on candidate variants, with sparse findings. We aimed to perform the first mitochondrial genome-wide association study of migraine, examining both single variants and mitochondrial haplogroups.

**Methods:**

In total, 71,860 participants from the population-based Nord-Trøndelag Health Study were genotyped. We excluded samples not passing quality control for nuclear genotypes, in addition to samples with low call rate and closely maternally related. We analysed 775 mitochondrial DNA variants in 4021 migraine cases and 14,288 headache-free controls, using logistic regression. In addition, we analysed 3831 cases and 13,584 controls who could be reliably assigned to a mitochondrial haplogroup. Lastly, we attempted to replicate previously reported mitochondrial DNA candidate variants.

**Results:**

Neither of the mitochondrial variants or haplogroups were associated with migraine. In addition, none of the previously reported mtDNA candidate variants replicated in our data.

**Conclusions:**

Our findings do not support a major role of mitochondrial genetic variation in migraine pathophysiology, but a larger sample is needed to detect rare variants and future studies should also examine heteroplasmic variation, epigenetic changes and copy-number variation.

## Introduction

It is well known that migraine tends to run in families, and twin studies examining migraine have estimated a heritability component of about 45% (1). Genome-wide association studies (GWAS) have found 38 migraine risk loci in nuclear DNA (nDNA) (2); however, these loci only explain about 15% of the total heritability (3). The remaining heritability may be explained by variants not yet examined, including variants in mitochondrial DNA (mtDNA).

The human mitochondrial genome is a 16,596 base pair long, circular structure, which is maternally inherited (4). It contains 37 genes, whereof 13 encode proteins involved in the respiratory chain, and the remaining encode ribosomal RNAs and transfer RNAs involved in the translation process (4). However, most of the > 1000 mitochondrial proteins are encoded by nDNA (5). Each cell contains hundreds to thousands of copies of the mtDNA, and there may be genetic variation between mtDNA molecules within the same cell, a phenomenon termed heteroplasmy (4).

The best-studied mutations in mtDNA are those that cause a critical energy failure, resulting in so-called ‘primary mitochondrial disorders’, such as Leber hereditary optic neuropathy (LHON), neurogenic muscle weakness, ataxia and retinitis pigmentosa (NARP), mitochondrial encephalopathy, lactic acidosis and stroke-like episodes (MELAS) and myoclonic epilepsy with ragged-red fibers (MERRF) (4). Studies of varying quality have also reported associations between mtDNA variation and several complex disorders, including cardiovascular disease, Alzheimer’s disease and Parkinson’s disease (4), as well as metabolic traits such as body weight and insulin levels (6).

Several lines of evidence indicate that mitochondrial dysfunction is involved in migraine pathogenesis. Findings of structurally abnormal mitochondria and signs of impaired energy metabolism have been reported in migraine patients, with similar patterns seen in some of the primary mitochondrial disorders, including MELAS, MERRF and LHON (7,8). In addition, a migraine-like headache is seen in several mitochondrial disorders, most commonly in MELAS (5,8). Lastly, migraine seems to be disproportionally inherited through the maternal line (9), consistent with a partial mitochondrial inheritance.

Until now, studies of mtDNA and migraine have focused on a few variants, and mainly rare mutations known to be associated with the mitochondrial encephalomyopathies, including MELAS, MERRF, Kearns-Sayre syndrome (KSS) and LHON. The studies are of small sample sizes, and none of the variants associated with the mitochondrial encephalomyopathies have shown associations with migraine (5,7,8). A few other studies have reported associations with other mtDNA variants. The largest study included 120 migraine patients and reported associations at m.16519C>T and m.3010G>A (10). Another study found an associated variant at m.4336A>G (11).

Candidate gene association studies are prone to give false positive findings (12), and no mitochondrial genome-wide association study of migraine has been performed as of today. We aimed to explore whether migraine was associated with variants across the mitochondrial genome, and with mitochondrial haplogroups. In addition, we aimed to replicate previously reported associations.

## Methods

### The Nord-Trøndelag Health Study

The Nord-Trøndelag Health Study (HUNT) is a large, population-based cohort study from Nord-Trøndelag county in Norway. The study has been carried out four times (HUNT1–4), and all inhabitants aged ≥ 20 have been invited to participate (13). Data was collected through questionnaires including more than 200 health-related questions and clinical examinations. DNA from whole blood was collected in HUNT2 (1995–1997) and HUNT3 (2006–2008), with genotypes being available in 71,860 participants.

**Figure 1.**
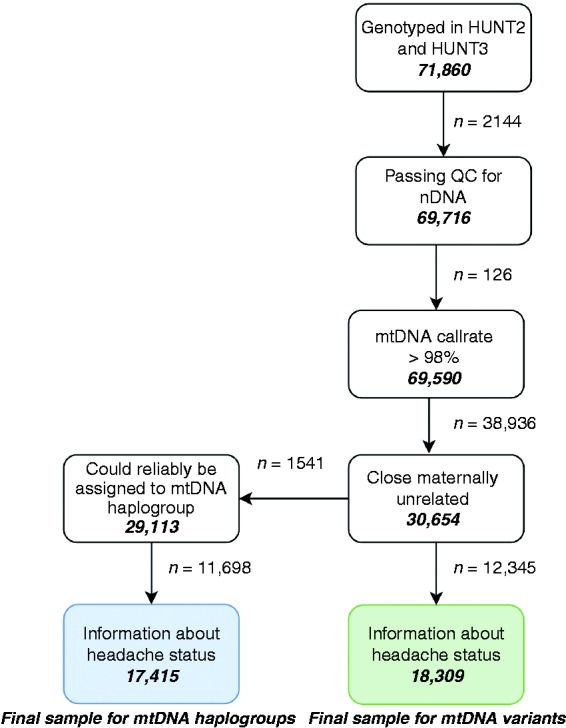
Overview of sample quality control and phenotype assignment. HUNT: the Nord-Trøndelag Health Study; QC: quality control; nDNA: nuclear DNA; mtDNA: mitochondrial DNA.

An overview of sample quality control and phenotype assignment is shown in Figure 1.

### Genotyping

Genotyping of 71,860 participants was performed at the Genomics-Core Facility (GCF) at the Norwegian University of Science and Technology, NTNU by the HUNT-Michigan (HUNT-MI) collaboration. Three different versions of the Illumina HumanCoreExome microarray were used (Illumina HumanCoreExome12 v.1.0, HumanCoreExome12 v.1.1 and HumanCoreExome24 with custom content), which included 369–394 mitochondrial variants.

#### Mitochondrial genotypes

**Figure 2.**
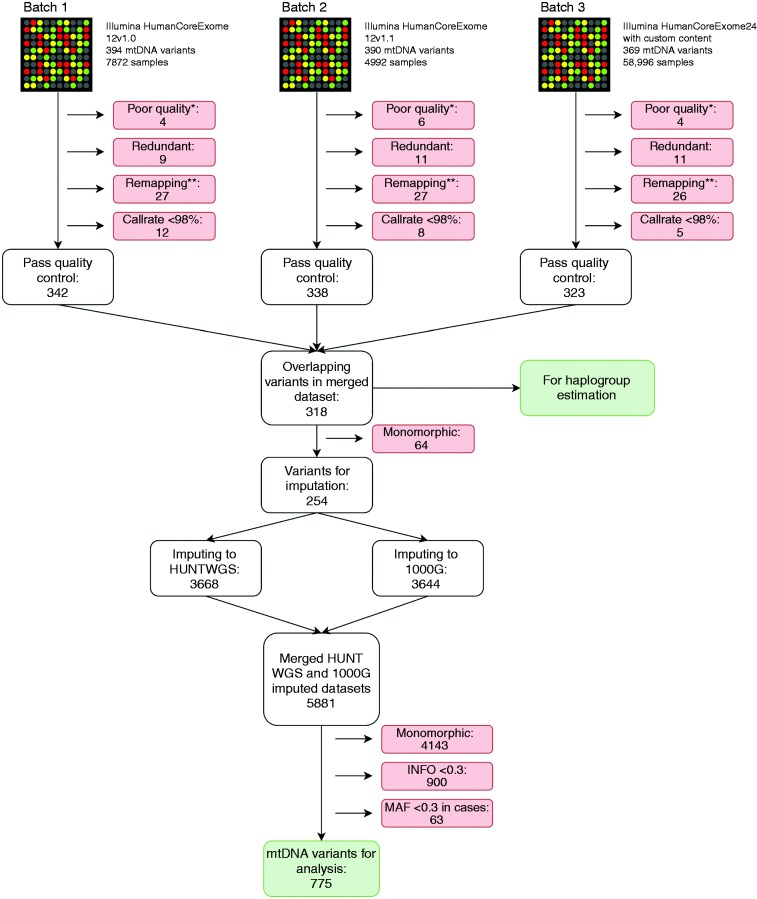
Overview of calling, quality control and imputation of mitochondrial genotypes. *Poor quality: Poor quality of the clusters in Illumina GenomeStudio, determined by visual inspection. **Remapping: Excluding variants with bad probe, multiple matches in genome and single mismatch. mtDNA: mitochondrial DNA; WGS: whole genome sequenced; INFO: imputation quality score; MAF: minor allele frequency.

An overview of the calling, genotyping and imputation of the mitochondrial variants is provided in Figure 2. The mitochondrial genotypes were called in Illumina GenomeStudio v.2011.1. All variants were manually clustered, allowing only homozygous calls. Heteroplasmic observations falling between the two homozygous clusters were excluded. Variants with low quality, as determined by visual inspection, redundant variants of the lowest quality and variants with call rate < 98% were excluded. To determine genomic position, strand orientation and reference allele, the genotypes were aligned to the Cambridge Reference Sequence (rCRS) using BLAT (14), and only variants that could be mapped perfectly, or with unique match with a single mismatch, were included. We excluded samples not passing quality control for nuclear genotypes, as described elsewhere (15) or with call rate < 98% for mitochondrial genotypes. After quality control, the datasets were merged, and only overlapping variants kept. All overlapping variants had comparable allele frequencies. Prior to imputation, monomorphic variants were removed. To allow haploid imputation, we assigned all samples to male sex, and followed instructions for imputing chromosome X in IMPUTE2 (16). We used a combination of 1000 Genomes Project (17) and 2191 low-coverage whole-genome sequenced samples from the HUNT study as reference panels, imputing to 5881 variants. Variants with imputation quality (INFO) score < 0.3 and monomorphic variants were excluded. The posterior probabilities from IMPUTE2 were converted to dosages, ranging from 0–1.

Since there are currently no established methods to handle mitochondrial relatedness, we excluded first- and second-degree maternally related samples. Relatedness was estimated from nuclear genotypes using KING (18), and maternal relatedness was defined as having zero or one mismatching mtDNA variant, as calculated with the –genome full command in PLINK 1.9 (19). After quality control, we had a sample of 30,564 participants who were not closely maternally related.

#### Mitochondrial haplogroups

Mitochondrial haplogroups were estimated from the directly genotyped variants, using PhyloTree17^20^ as implemented in HaploGrep2 (21). We assigned participants to one of the major European haplogroups (H, V, HV, J, T, U, K, Z, W, X, I, N), or to a group of “Others”. Samples with a haplogroup quality score < 0.9 were excluded, in addition to those failing quality control for mitochondrial or nuclear genotypes, or who were closely maternally related, resulting in a sample of 29,113 participants.

### Migraine diagnosis

The migraine diagnosis was assessed using questionnaires and based on a modified version of the International Classification of Headache Disorders (ICHD) (22). The participants were asked whether they had suffered from headache during the last 12 months, and those who answered “yes” were classified as headache sufferers, while those who answered “no” constituted the control group of headache-free individuals. The headache sufferers were asked subsequent questions about their headache, and were classified as having migraine if they fulfilled the following three criteria: (a) Headache attacks lasting 4–72 hours (<4 hours was accepted for those who reported commonly occurring visual disturbances before headache); (b) headache with at least one of the following characteristics: Pulsating quality, unilateral location, or aggravation by physical activity; (c) during headache, at least one of the following occurred: Nausea, photophobia or phonophobia. In addition, the participants were asked if they suffered from migraine; those who responded positively to this question were also included in the migraine group. The migraine diagnoses were validated by clinical interviews performed by neurologist. In HUNT2, the sensitivity was 69% and specificity 89% (κ = 0.59, 95% CI 0.47–0.71) (23). In HUNT3, the sensitivity was 49% and specificity was 96% (κ = 0.51, 95% CI 0.24–0.68) (24). The final sample sizes are given in Figure 1.

### Covariates

Covariates included in the analyses were sex, birth year, genotype batch and first four principal components. The principal components were calculated from nuclear genotypes by projecting all samples into the space of the principal components of the Human Genome Diversity Project (HGDP) reference panel, using PLINK v1.90 (19).

### Statistical analysis

#### Mitochondrial variants

**Figure 3.**
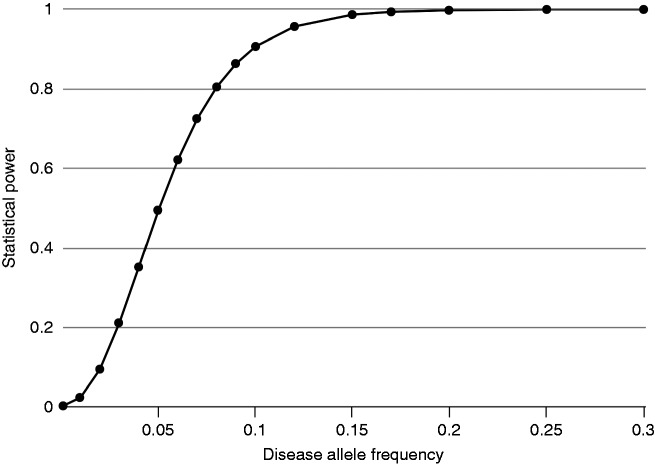
Plot of statistical power versus disease allele frequency, assuming an additive model, disease prevalence of 0.14 and genotype relative risk of 1.2.

We performed a logistic regression, using the Firth test in EPACTS v3.3.0 (25). Migraine was modelled as the dependent variable, and the genotyped variants (where available) or imputed variants (dosages) as the independent variable. Sex, birth year, batch and first four principal components were included as covariates. Only variants with an estimated minor allele count (MAC) ≥ 3 in the case group were included, which equalled a minor allele frequency (MAF) threshold of 0.00075. We performed a spectral decomposition analysis (26) to determine the number of independent genetic effects, which was 249. Using Bonferroni correction, the adjusted significance threshold was *p* ≤ 0.05/249 ≤ 2.005 × 10^−4^. A post-hoc power calculation was performed using the Genetic Association Study (GAS) Power calculator (27). Assuming an additive model, migraine prevalence of 0.14 and genotype relative risk of 1.2 (for carriers vs. non-carriers of the risk allele), we had 80% power to detect risk variants with MAF > 0.08. The power to detect an effect of this magnitude will be progressively lower for variants rarer than this (Figure 3).

In order to evaluate previous studies, we specifically examined variants reported to be associated with migraine, or that are strongly implied in the mitochondrial encephalomyopathies MELAS, MERRF, KSS and LHON (5,7,8). Some of the rare variants were not present in our dataset, and six variants were tested (see Supplemental Table 1). We used a Bonferroni-corrected *p*-value threshold ≤ 0.05/6 ≤ 0.008 to indicate significance. Since the study from Zaki et al. (10) only included samples with haplogroup H, and since m.3010G > A was significant only in those with the m.16519C > T variant, we performed additional analyses in the same subgroups. As m.16519C > T was imputed, we defined the presence of a T allele as having a dosage of > 0.5.

#### Mitochondrial haplogroups

The candidate haplogroup was analysed by using all the other haplogroups as reference. We performed a logistic regression, using Firth test in EPACTS v3.3.0 (25). Sex, birth year and batch were included as covariates. We did not include principal components, as haplogroup and principal components are likely to be correlated, both expressing information about ancestry. By applying Bonferroni correction, the adjusted *p*-value threshold was ≤ 0.05/13 ≤ 0.0038.

### Ethics

Participation was based on informed, written consent, and the study was approved by the Regional Committee for Medical and Health Research (#2015/576/REK Midt and #2014/144/REK Midt). In addition, the HUNT Study was approved by the Norwegian Data Inspectorate. The HUNT biobank is approved by REK (#4/2006/250/REK Midt).

## Results

### Mitochondrial variants

**Figure 4.**
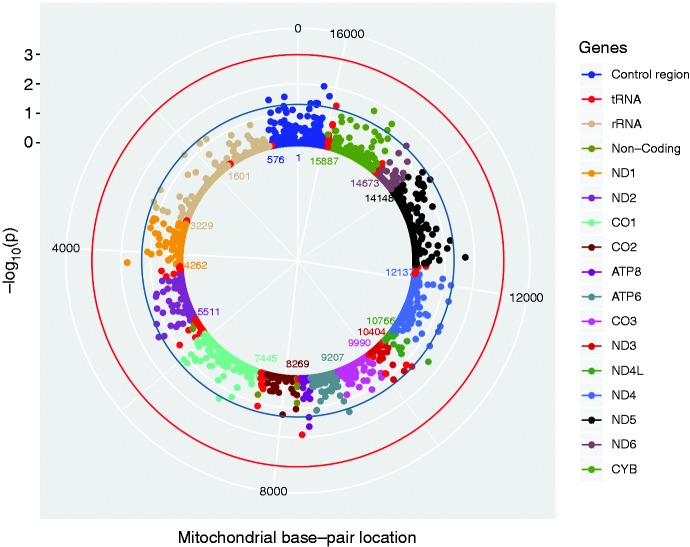
Solar Manhattan plot of the association *p*-values between mitochondrial DNA (mtDNA) variants and migraine. Each dot represents a mtDNA variant association with migraine, colour-coded by gene. The basepair positions are provided on a circular x-axis, anticlockwise, to represent the structure of the mtDNA. The - log10(*p*)-values are provided on a radial y-axis, which is the distance from the centre. The red circle indicates the Bonferroni-corrected *p*-value threshold of 2.005 × 10^-4^. The blue line indicates *p* = 0.05.

**Figure 5.**
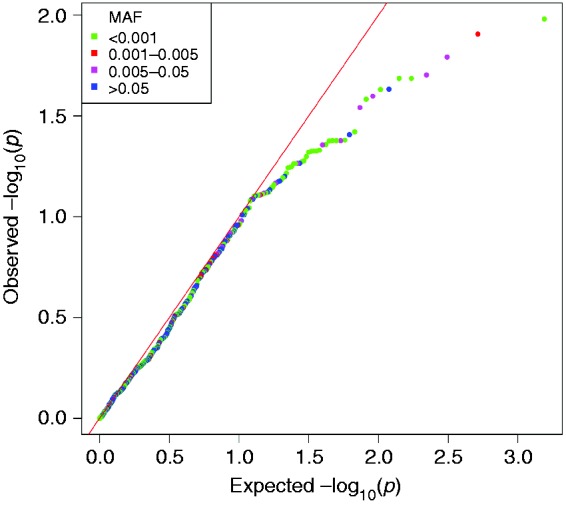
Quantile-quantile plot of association *p*-values between mitochondrial DNA variants and migraine. The x-axis represents the expected -log10(*p*)-values, and the y-axis represents the observed -log10(*p*)- values. The red line represents the distribution of *p*-values under the null. Variants are coloured by minor allele frequency (MAF).

For the analysis of mtDNA variants we included 18,309 participants; 4021 cases and 14,288 controls. Of the 775 variants that were analysed, none were significantly associated with migraine after correction for multiple testing (Figures 4 and 5). A moderate deviation from the expected association results, seen as a deflation in the quantile-quantile (QQ)-plot (Figure 5), indicates that our *p*-values are higher than would be expected by chance. As the mtDNA is a small genome with highly correlated variants, it is possible that the deflation reflects stochastic variation due to the limited number of independent effects.

For the replication of variants that have previously been associated with migraine, or with mitochondrial encephalomyopathies, none were significantly associated with migraine in our data (Supplemental Table 1).

### Mitochondrial haplogroups

We analysed mitochondrial haplogroups in 17,415 participants; 3831 cases and 13,584 controls. The distribution of the haplogroups (see Table 1) agreed with previous reports from the Norwegian population (28). None of the haplogroups were significantly associated with migraine (Table 1).

**Table 1.** Association results between mitochondrial haplogroups and migraine.

Haplogroup	Freq	Beta	SE Beta	*p*-value	Freq cases	Freq controls
H	0.40	0.03	0.04	0.48	0.40	0.40
V	0.02	−0.08	0.13	0.53	0.02	0.02
HV	0.05	0.01	0.09	0.92	0.05	0.05
J	0.15	−0.02	0.05	0.70	0.15	0.16
T	0.08	−0.02	0.07	0.78	0.08	0.08
U	0.17	−0.05	0.05	0.36	0.16	0.17
K	0.09	−0.01	0.07	0.94	0.09	0.09
Z	0.01	0.12	0.25	0.65	0.01	0.01
W	0.01	0.16	0.16	0.31	0.02	0.01
X	0.01	0.00	0.19	1.00	0.01	0.01
I	0.01	0.27	0.17	0.11	0.01	0.01
N	0.00	−0.45	0.61	0.43	0.00	0.00
Other	0.00	0.61	0.52	0.24	0.00	0.00

Freq: haplogroup frequency; SE: standard error.

## Discussion

### Summary of the main findings

In this first mitochondrial genome-wide association study of migraine, we found no associations with mtDNA variants or haplogroups. Furthermore, no significant associations were found when specifically considering previously reported associated variants, and variants known to be associated with the migraine-related mitochondrial encephalomyopathies. Thus, we did not find evidence for a major role of mitochondrial genetic variation in migraine.

### Consistency with previous studies

Previous studies on mitochondrial genetic variation in migraine have mainly focused on candidate variants that are strongly implied in the mitochondrial encephalomyopathies. None of these variants have shown associations with migraine (5,7,8).

A few studies have reported positive findings for variants not linked to the mitochondrial encephalomyopathies. Zaki et al. (10) initially analysed patients with cyclic vomiting syndrome, which is a migraine equivalent that was used as a model disorder because of having less heterogeneity. They found two associated variants, which were further analysed in 112 migraine without aura cases and 444 controls. The variant m.16519C>T was significantly associated with migraine, and m.3010G>A was significant in those with the m.16519C>T polymorphism, suggesting a synergistic effect. Despite a substantially larger sample size, we could not confirm these findings in our sample of migraine patients. The reported associations may have resulted from different ancestry between the cases (from Germany) and controls (from USA, Britain, Italy and Finland) (10). Another study that analysed the variant m.4336A>G and a variety of diseases, reported an association with migraine (11). However, the sample size was small, and only two out of 42 migraine cases harboured the variant. In our sample of 4021 migraine cases, we did not replicate this finding.

The fact that we could not replicate these previous findings is in line with previous studies, which have shown that candidate gene association studies seem prone to false positives. This may be explained by a low probability of targeting the causal variant, small sample sets, and lack of robust replication in independent cohorts (12). As an example, one study re-evaluated 27 genes in nuclear DNA where associations to migraine had previously been found in candidate gene studies and other non-GWA studies. None of the previous findings replicated in a large GWAS dataset comprising 5175 migraine cases and 13,972 controls (12). Similar experiences are also reported for other common diseases; for example, psychiatric diseases (29). In contrast to candidate gene studies, genome-wide studies, are hypothesis free with regards to causative genes, typically involve a much larger sample and apply stringent corrections for multiple testing.

### Methodological considerations

#### Accounting for relatedness in analysis of mtDNA

The fact that mtDNA is maternally inherited has implications for how mtDNA should be analysed in population-based association studies. We believe that the assumption about independent observations is only violated for close maternal relatives, because the observations are dependent both in terms of exposure (genetic variants) and outcome (migraine). Even close paternal relatives do not share more mtDNA than the general population and are therefore independent in terms of exposure. Distant maternal relatives share mtDNA, but do not share other factors that may affect the risk of developing migraine, including nuclear variants and environmental factors, and are therefore independent in terms of outcome. Furthermore, since mtDNA does not recombine, but behaves like a single locus with many alleles (30), all variants are more or less correlated with each other. Adjusting for all maternal relatedness (e.g. all individuals with identical, or almost identical mtDNA), would result in fitting the candidate variant twice into the model. A comparable situation occurs in traditional GWAS, where the whole chromosome containing the candidate variant is often excluded from calculation of the genetic relationship matrix, in a method called “leave-one-chromosome-out” (LOCO), to avoid fitting the candidate variant twice and losing power (31). To our knowledge, no adequate methods currently exist for adjusting for close maternal relatedness in mitochondrial association studies.

#### Strengths and limitations

Strengths of the study include a large and unselected population. Cases and controls were genotyped on the same array and have been handled together independent of the phenotype studied, excluding systematic batch effects between the two.

Limitations include that the migraine diagnosis was based on questionnaires, rather than clinical interviews by experienced physicians. However, the diagnosis was based on the ICHD criteria, and has been validated by interviews by neurologists, yielding high specificity for migraine (23,24). Only current migraine, present during the last 12 months, was assessed. While this reduces the risk of recall bias, it also increases the risk of including previous migraine sufferers, who are currently headache free, into the control group. This may lead to some deflation of our results.

#### Unanswered questions

While we only examined inherited, homoplasmic mutations present in blood, there may be other types of variation that could play a role in migraine pathogenesis.

Heteroplasmy refers to mtDNA variants that are present in only a fraction of the mtDNA molecules (4). Heteroplasmic variants may result from somatic mutations, or they may be maternally inherited. Recent studies have found heteroplasmies in almost all individuals, although in small amounts (32). Additionally, many heteroplasmies, including the inherited ones, seem to be tissue specific (33). Ideally, mtDNA variation should be examined in relevant tissue, but the type of tissue or cell that would be relevant in migraine is not clear. To capture low-level heteroplasmy, sensitive sequencing methods are needed (34).

While we examined both common and rare variants, larger studies are needed to detect uncommon variants with low to moderate effects. Sequencing will be required for a complete coverage of rare variants, including those not present in the imputation reference panels. Other forms of genetic variation, such as epigenetic changes or copy number variation, also remain to be examined. Mitochondrial function is also affected by variants in nuclear genes encoding mitochondrial proteins, which have not been covered in the present study. The largest, and most recent GWAS on migraine did not specifically report any findings in genes encoding mitochondrial proteins (2), but a more specific examination of these regions should be performed in the future. Lastly, we have performed our analyses in samples of European ancestry only, and our findings can not necessarily be transferred to other populations.

## Conclusion

In this first mitochondrial-wide association study of migraine, we did not find association to any mitochondrial variant or mitochondrial haplogroup. Despite epidemiological studies supporting a role of mitochondrial genetic variation in migraine, our large study did not confirm this. Still, a larger sample will be needed to detect rare variants. Future studies should also examine heteroplasmic variation, epigenetic changes, copy-number variation and variation in nuclear genes encoding mitochondrial proteins.

## Supplemental Material

CEP906835 Supplemental table 1 - Supplemental material for Mitochondrial genome-wide association study of migraine – the HUNT Study Click here for additional data file. Supplemental material, CEP906835 Supplemental table 1 for Mitochondrial genome-wide association study of migraine – the HUNT Study by Sigrid Børte, John-Anker Zwart, Anne Heidi Skogholt, Maiken Elvestad Gabrielsen, Laurent F Thomas, Lars G Fritsche, Ida Surakka, Jonas B Nielsen, Wei Zhou, Brooke N Wolford, Magnus D Vigeland, Knut Hagen, Espen Saxhaug Kristoffersen, Dale R Nyholt, Daniel I Chasman, Ben M Brumpton, Cristen J Willer and Bendik S Winsvold in Cephalalgia

## Key findings

- No variants in mitochondrial DNA mitochondrial haplogroups were found to be associated with migraine.
- None of the previously reported associations between mitochondrial DNA and migraine replicated in our data.
- Mitochondrial variation not covered by this study remain to be examined, including rare and heteroplasmic variants, epigenetic changes, copy-number variation and variation in nuclear genes encoding mitochondrial proteins.
