# Supplementary material for: Mitochondrial genome-wide association study of migraine – the HUNT Study
Source: Cephalalgia. 2020 Feb 14;40(6):625–34. doi: 10.1177/0333102420906835 (PMC7243449; doi:10.1177/0333102420906835)
Supplement: CEP906835 Supplemental table 1 - Supplemental material for Mitochondrial genome-wide association study of migraine – the HUNT Study [file CEP906835_Supplemental_table_1.pdf]

Suppl. Table 1. Association results between mitochondrial DNA variants previously reported to be associated with migraine or mitochondrial encephalomyopathies, and migraine.

Variant	Associated with	Beta	SE beta	P-value	MAF
m.4336A>G	Migraine	−0.16	0.13	0.23	0.02
m.16519C>T	CVS, migraine				
-All		-0.04	0.04	0.32	0.41
-Haplogroup H		-0.07	0.05	0.15	0.48
m.3010G>A	CVS, migraine				
-All		0.03	0.04	0.45	0.28
-Haplogroup H		-0.01	0.06	0.82	0.22
-With 16519C>T		-0.01	0.06	0.87	0.13
-Haplogroup H with 16519C>T		0.02	0.07	0.81	0.17
m.11084A>G	MELAS	−0.33	0.41	0.41	0.01
m.4216T>C	LHON	−0.01	0.05	0.76	0.22
m.13708G>A	LHON	0.00	0.05	0.99	0.16

MAF: Minor allele frequency, CVS: Cyclic vomiting syndrome, MELAS: Mitochondrial encephalomyopathy, lactic acidosis, and stroke-like episodes, LHON: Leber hereditary optic neuropathy. The following, rare variants were not present in our data, either because they were monomorphic (and thus excluded), or they were not present in the reference panels: m.3243A>G and m.3271T>C (MELAS); m.8344G>A (MERRF); deletion on 4,977 (KSS); pos 14,484, pos 11,778, pos 3,460 and pos 4,160 (LHON primary).
